# Effects of Two Culture Modes on Muscular Nutrition Content and Volatile Flavor in Chinese Longsnout Catfish (*Leiocassis longirostris*)

**DOI:** 10.3390/biology14060694

**Published:** 2025-06-13

**Authors:** Luo Zhou, Yingbing Su, Daiqin Yang, Qiong Shi, Tilin Yi, Zhengyong Wen

**Affiliations:** 1 School of Animal Science, Yangtze University, Jingzhou 424020, China; zhouluo3211@163.com (L.Z.); su-yingbing@163.com (Y.S.); yangdaiqin319@163.com (D.Y.); 2 Key Laboratory of Sichuan Province for Fishes Conservation and Utilization in the Upper Reaches of the Yangtze River, Neijiang Normal University, Neijiang 641100, China; shiqiong@szu.edu.cn; 3 College of Life Sciences, Neijiang Normal University, Neijiang 641100, China; 4 Shenzhen Key Lab of Marine Genomics, BGI Academy of Marine Sciences, Shenzhen 518081, China; 5 Laboratory of Aquatic Genomics, College of Life Sciences and Oceanography, Shenzhen University, Shenzhen 518057, China

**Keywords:** longsnout catfish, culture mode, nutritional composition, volatile compound

## Abstract

Thus far, various aquaculture modes have been developed to facilitate the rapid growth of the aquaculture industry and thus meet the heavy demand for aquatic products for human consumption. However, the effects of different culture modes on fish muscular nutritional content and volatile flavor are rarely reported. In the present study, we evaluated the differences in muscular nutrition content and dietary flavor between Chinese longsnout catfish (*Leiocassis longirostris*) groups cultured in two different modes, i.e., flow-through water tanks (CWWL) and traditional ponds (CWWC). Our statistical results showed that a significantly higher crude protein content and lower crude fat levels were observed in the CWWL group than in the CWWC group (*p* < 0.05). Similarly, the contents of total aromatic amino acids (Total ∑TAA) and total dicarboxylic amino acids (Total ∑DAA) were also significantly higher in the CWWL group. Among the fatty acids, long-chain polyunsaturated fatty acids (LC-PUFAs), including eicosapentaenoic acid (EPA), docosahexaenoic acid (DHA), and arachidonic acid (ARA), were recorded at 1.44%, 2.5%, and 9.09%, respectively, in the CWWL group, which were dramatically higher than in the CWWC group. Conversely, the contents of volatile compounds, including 2-tridecanone, dimethyl trisulfide, and isophorone, in the CWWC group were also significant higher, which, however, may contribute to an unpleasant sensory experience. Conversely, other compounds like 2-methylbutanal and 2,3-butanedione were prevalent in the CWWL group, which can induce rich nutty and buttery flavors and thus enhance the freshness of flavor profiles. In conclusion, Chinese longsnout catfish cultured in flow-through tanks show higher nutritional value and better sensory flavor in comparison with those raised in ponds. These findings not only provide novel insights into the potential effects of aquaculture modes on muscular nutrition content and dietary flavor for Chinese longsnout catfish but also lay a solid foundation for optimizing practical culture modes to improve the global aquaculture industry.

## Simple Summary

Aquaculture modes significantly influence the nutritional and flavor profiles of farmed fish. This study compared Chinese longsnout catfish (*Leiocassis longirostris*) cultured in flow-through water tanks (CWWL) to those raised in traditional ponds (CWWC). Interestingly, the CWWL group exhibited significantly higher crude protein content (*p* < 0.05) and lower fat levels than the CWWC group. Conversely, the CWWC group had higher levels of volatile compounds (such as 2-tridecanone and dimethyl trisulfide) linked to unpleasant odors, while the CWWL group showed increased 2-methylbutanal and 2,3-butanedione concentrations, which enhance nutty and buttery flavors. These findings demonstrate that flow-through tank culture improves nutritional quality (such as higher contents of protein and LC-PUFAs) and sensory attributes compared to pond systems.

## 1. Introduction

Chinese longsnout catfish (*Leiocassis longirostris*), belonging to the family Bagridae and the order Siluriformes, are carnivorous demersal fish that are primarily distributed in the Yangtze, Huaihe, and Pearl rivers in China [1]. This fish species is welcome by consumers due to its several valuable traits, including no intermuscular spines, high nutritional value, and good taste [2]. As a result, it has been listed in the “Four famous fresh fishes in the Yangtze river” [3]. However, the wild population of Chinese longsnout catfish has significantly declined in recent years due to environmental disruption, water pollution, dam construction, and overfishing [4]. In this context, artificial breeding and the release of cultured offsprings into wild rivers are effective strategies to recover the wild population [5].

Thus far, studies of Chinese longsnout catfish have primarily focused on its biological characteristics, reproduction and breeding technologies, nutritional requirements, culture modes, and disease management. To date, three categories of culture modes, including pond culture, recirculating aquaculture system (RAS) culture, and net-cage culture, have been widely developed in China [6]. Among them, pond culture is the most substantial way to obtain Chinese longsnout catfish, but this mode faces serious challenges, such as frequent disease outbreaks, excessive drug residues, and diminished fish quality [7]. Conversely, the RAS is an emerging mode, where wastewater from the ponds is treated and re-circulated through a series of water treatment units. This has several supportive advantages, including high yield and quality, easy management, and lower drug residues [8]. Therefore, RAS culture has been recognized as an economically viable and environmentally sustainable method, and it has been widely applied for the cultivation of valuable economic fishes such as tilapia, grass carp, and rainbow trout [9].

A previous study showed that the aquaculture environment significantly influences fish nutritional content and dietary flavor [10]. This work encompasses a comparative analysis of morphological characteristics and nutritional quality among different aquaculture systems. For largemouth bass (*Micropterus salmoides*), fishes cultured in land-based RASs significantly improved the contents of crude protein, docosahexaenoic acid (DHA), and fresh amino acids when compared with those cultured in traditional ponds [11]. Similarly, the nutritional values and volatile flavor compounds of common carp (*Cyprinus carpio*) were significantly higher in an in-pond runway system or wild environment than for fish cultured in traditional ponds [12]. For bighead carp (*Aristichthys nobilis*), fish cultured in a common pond exhibited lower myofiber density and dietary taste than those cultured in natural lakes or cold-water reservoirs [13]. Consistently, another study revealed that grass carp (*Ctenopharyngodon idella*) in recirculating pond aquaculture systems (RPASs) showed a higher growth rate, higher levels of aromatic amino acids, and more freshness flavor compounds in the muscles [14]. In summary, these reports demonstrate that aquaculture modes have differential effects on the nutritional value and dietary flavor of fish meat, and thus optimizing a suitable culture mode will be beneficial for improving nutritional value and dietary flavor in cultured fishes.

**Figure 1:**
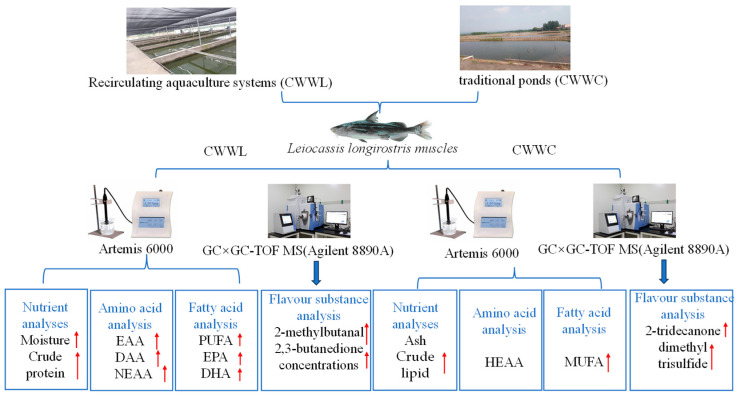
Workflow for this study. The arrows represent up regulation or higher content in the two culture systems.

In this study, we focused on Chinese longsnout catfish, with the primary goal of evaluating the overall effects of the individual culture mode (traditional pond culture, CWWC, or recirculating water culture, CWWL) as an integrated production system on fish muscle nutrient composition and volatile flavor compounds. By systematically comparing the differences in fish quality between these two modes, we try to determine which mode is more favorable to improve the overall quality of this valuable fish (Figure 1). Our findings will not only provide more details about the nutritional content and dietary flavor composition of the Chinese longsnout catfish raised in the two different systems but will also lay a solid foundation for the practical selection of an appropriate culture mode for this economic fish species.

## 2. Materials and Methods

### 2.1. Fish Sampling

This experiment was conducted at a local aquaculture base for Chinese longsnout catfish in Dangyang city, Hubei province, China. In brief, a batch of Chinese longsnout catfish larvae (average bodyweight of 500 g) were randomly assigned and cultured in an RAS (CWWL) or traditional pond (CWWC) environment. The culture density was set as 15,000 tails in the RAS (22 × 5 × 2.5 m) and 15,000 tails per hectare in the ponds. These experimental fish were fed with commercial feed at 3–5% of their average bodyweight twice daily (8:00 and 18:00). The culture period last for 8 months, and then three fish (average weight of 1.5 kg) were randomly selected for sampling from the RAS and the pond, respectively.

The fish were fasted for 24 h before sampling, and then they were anesthetized with MS222 (China, Changsha Shanghe Biotechnology Co., Changsha, China) before dissection. After that, dorsal muscle tissues from the corresponding locations on both sides of each fish body were removed and placed into two sampling tubes, of which one was used for the measurement of general nutrition contents, and the other was used for the detection of dietary volatile compounds. All tissues were frozen in liquid nitrogen, and three replications of these fish raised in the two culture modes were conducted in all experiments. The animal study protocol was approved by the Ethics Committee of Yangtze University (protocol code DKXY2024016).

### 2.2. Determination of Nutrient Composition

The gross chemical composition was analyzed following standard methods provided by the Association of Official Analytical Chemists (AOAC) [15]. In short, moisture content was detected by direct drying at 105 °C (GB/T 6435-2014) [16], crude ash was measured with a muffle furnace volatile constant-weight method (GB/T 6438-2007) [17], crude fat content was determined by using the Soxhlet extraction method (GB 5009.6-2016) [18], and crude protein was determined by using the micro-Kjeldahl method (GB 5009.5-2016) [19]. Amino acid composition was evaluated according to the food safety national standard of China (GB/T 18246-2019) [20]. The fatty acid composition of the muscles was determined by gas chromatography, and the fatty acid content of each component was calculated by an area normalization method (GB 5009.168-2016) [21]. Detailed parameters are set as follows. fatty acid methyl esters (FAMEs) were separated using an Agilent HP-88 capillary column (Agilent Technologies, Santa Clara, CA, USA) (100 m × 0.25 mm × 0.20 μm) under programmed temperature conditions: initial hold at 140 °C for 5 min, followed by a 4 °C/min ramp to 240 °C with a 15 min final hold. Helium carrier gas was delivered at a constant flow rate of 1.0 mL/min. The injector and flame ionization detector (FID) were maintained at 250 and 260 °C, respectively, with a 10:1 split ratio.

### 2.3. Determination of Dietary Flavor

Volatile compounds were separated and detected by using gas chromatography–mass spectrometry (GC-MS) [22]. A comprehensive two-dimensional GC × GC-TOF MS chromatographic system was combined with an Agilent 8890A gas chromatograph (Agilent Technologies) (with high-purity helium as the carrier gas) at a constant flow rate of 1.0 mL/min. Detailed steps for the one-dimensional chromatographic column were designed as follows: the initial temperature was maintained at 40 °C for 5 min, then elevated to 100 °C at a rate of 5 °C/min, and then increased to 120 °C at a rate of 2 °C/min, held for 3 min, and finally increased to 250 °C at a rate of 6 °C/min. The heating procedure of the 2D column was set at 5 °C higher than that of the 1D column, the modulator temperature was always set at 15 °C higher than that of the 2D column, and the modulation period was set as 4.0 s. The temperature of the inlet and sample port was maintained at 250 °C.

LECO Pegasus BT 4D mass spectrometry (LECO Corporation, Burghausen, Germany) was run under the conditions of a mass spectrometry transmission line temperature of 250 °C, an ion source temperature of 250 °C, an acquisition rate of 200 spectra/s, an electron bombardment source of 70 eV, a detector voltage of 2020 V, and a mass spectral scanning range of 35–550 *m*/*z*.

### 2.4. Measurement of Major Volatile Compounds and Relative Contents

The odor activity value (OAV) method [23] was employed to evaluate major volatile compounds, i.e., the contribution of OAV = C/T, where C represents the concentration of an individual sensitizer and T is the threshold value perceived by the human nose. Since samples usually contain multiple volatile compounds, we applied the relative olfactory activity value (ROAV) to analyze the contribution of each compound to the total sample.

ROAVi ≈ 100 × Cri/Crmax × Tmax/Ti

Among them, Cri and Ti are the relative content of each volatile compound and the corresponding sensory threshold, respectively. Crmax and Tmax are the relative content of the component that contributes most to the overall flavor of each sample and the corresponding sensory threshold. The relative content of each compound was determined by the peak area normalization method [24].

### 2.5. Data Processing

The analysis of the flavor–odor value contribution based on untargeted flavoromics was performed by Panomics Biomedical Technology Co., Ltd. (Suzhou, China), based on reference data; preliminary identification was carried out by comparing the linear retention index (RI), 1- and 2-dimensional retention times, and mass spectra of the GC × GC-TOF-MS data with the NIST20 database. Substances were then classified and annotated using the Classyfire software (http://classyfire.wishartlab.com/) [25], and all flavor compounds were predicted from the FlavorDB database and the Odor database. Finally, multivariate statistical analyses were conducted, including principal component analysis (PCA), partial least-squares discriminant analysis (PLS-DA), and orthogonal projection to latent structure discriminant analysis (OPLS-DA). The two differential fish groups were analyzed according to the OPLS-DA score plots, and metabolite differences were screened according to the value of the contribution (VIP) versus significant difference (*p* < 0.05).

### 2.6. Statistical Analysis

All data for nutrient composition were expressed as mean ± standard deviation (SD). SPSS Statistics 26.0 was performed to analyze differences among groups. *p*< 0.05 indicates a significant difference.

## 3. Results

### 3.1. Summary of Basic Nutritional Content

The basic nutritional contents of dorsal muscles were detected and compared to investigate the potential effects of different culture modes on the nutrient composition of Chinese longsnout catfish. Our results showed that no significant difference in ash content was observed between the fish cultured in CWWC and CWWL (Table 1). However, the moisture and crude protein levels were significant higher in the CWWL group than in the CWWC group. Conversely, the crude lipid level was significant higher in the CWWC group than in the CWWL group (see Table 1).

### 3.2. Comparison of Amino Acid Composition

In general, a total of 17 categories of amino acids were identified in the dorsal muscle of Chinese longsnout catfish when cultured in both modes, including seven essential amino acids (EAAs: Lys, Phe, Met, Thr, Ile, Leu, Val), two semi-essential amino acids (HEAAs: His, Arg), and four flavor amino acids (DAAs: Glu, Asp, Ala, Gly). Among them, Glu was the most abundant, followed by Asp, Lys, and Leu (Table 2). Meanwhile, Cys was identified as the first limiting amino acid. The ratios of EAA/TAA and EAA/NEAA conformed to the ideal protein pattern, as defined by FAO/WHO, in both groups. Notably, the NEAA, EAA, and DAA levels were significantly higher in the CWWL group than the CWWC group (see more details in Table 2), although no significant difference was observed in HEAA levels between the two groups. The contents of flavor amino acids were also higher in the CWWL group than in CWWC group.

### 3.3. Differences in Fatty Acid Composition

A total of 24 fatty acids were detected in both groups, including 7 saturated fatty acids (SFAs: C12:0, C14:0, C15:0, C16:0, C17:0, C18:0, C20:0), 5 monounsaturated fatty acids (MUFAs: C14:1n5, C16:1n7, C17:1n7, C18:1n9c, C22:1n9), and 10 polyunsaturated fatty acids (PUFAs: C18:2n6c, C18:3n6, C18:3n3, C20:2, C20:3n6, C20:3n3, C22:2n6, AA, EPA, DHA). Interestingly, SFA and PUFA levels were significant higher in the CWWL group, whereas MUFA levels were significant lower compared to those in the CWWC group (Table 3).

C18:1n9c was the predominant fatty acid in both groups, followed by C16:0 and C18:2n6c, with C22:2n6 as the lowest fatty acid (Table 3). Notably, C18:1n9c and C18:2n6c were significantly higher in the CWWC group. Conversely, the concentrations of docosahexaenoic acid (DHA) and eicosapentaenoic acid (EPA) were significantly higher in the CWWL group.

### 3.4. Volatile Flavor Compounds

#### 3.4.1. Identification of Volatile Substances

**Figure 2:**
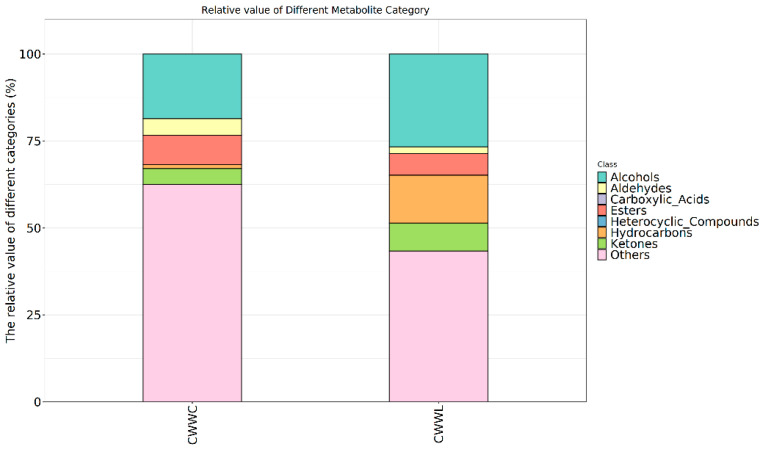
Relative content of volatile substance types in fish from both culture modes. Horizontal coordinates indicate sample groupings, vertical coordinates indicate the relative content of species, and colors indicate flavor substance types.

In this study, comprehensive profiling of volatile compounds was conducted by searching for the obtained data in the NIST database [26], and then they were classified with PubChem by using Classyfire software [27]. Our results showed that a total of 2000 volatile compounds were identified in the examined groups (Figure 2). The CWWC group contained 686 compounds, including 86 esters, 80 hydrocarbons, 61 alcohols, 41 ketones, 7 aldehydes, and 411 other compounds. The CWWL group was identified with 1314 compounds, including 304 hydrocarbons, 89 alcohols, 75 esters, 19 aldehydes, and 754 other compounds. Notably, no heterocyclic compounds or carboxylic acids were detected in either group, while both groups shared similarity in terms of the categories of compounds. Furthermore, the CWWL group included more enrich and diverse flavor profiles and exhibited significantly different major components in comparison with the CWWC group (see more details in Figure 2 and Table S1).

#### 3.4.2. Volatile Matter Threshold

**Figure 3:**
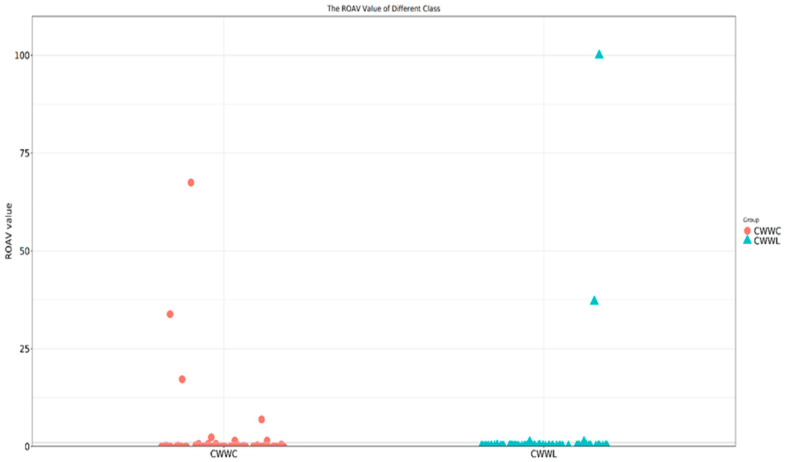
Scatter plot of ROAV odor activity values of Chinese longsnout catfish cultured under two modes. Horizontal coordinates indicate different groups, vertical coordinates indicate flavor substance ROAVs, and colors indicate different subgroups.

The Relative Odor Activity Value (ROAV) method was employed to evaluate the odor-active compounds in the muscle of Chinese longsnout catfish. Compounds with ROAV ≥ 1 were considered primary and key volatile flavor substances, whereas those with an ROAV between 0.1 and 1 were classified as auxiliary, and the latter enhanced the partial flavor profiles. In fact, in the CWWL group, the highest flavor contributors were 2-methylbutyraldehyde, 2,3-butanedione, 2-pentylfuran, and 2-undecane (Figure 3). These compounds produced prominent odors reminiscent of cocoa powder, almonds, and butter, along with notes of citrus, freshness, green beans, and vegetables. Conversely, the primary odor-active compounds in the CWWC group included 2-methylbutyraldehyde, 2-undecane, 2,3-butanedione, isophorone, and dimethyl trisulfide (see Figure 3). These compounds contributed to a range of odors from sharp and malodorous to oniony and pungent. Our findings distinctly illustrated the differences in the main components and overall flavor profiles between the CWWC and CWWL groups.

#### 3.4.3. Data on Multivariate Statistics

**Figure 4:**
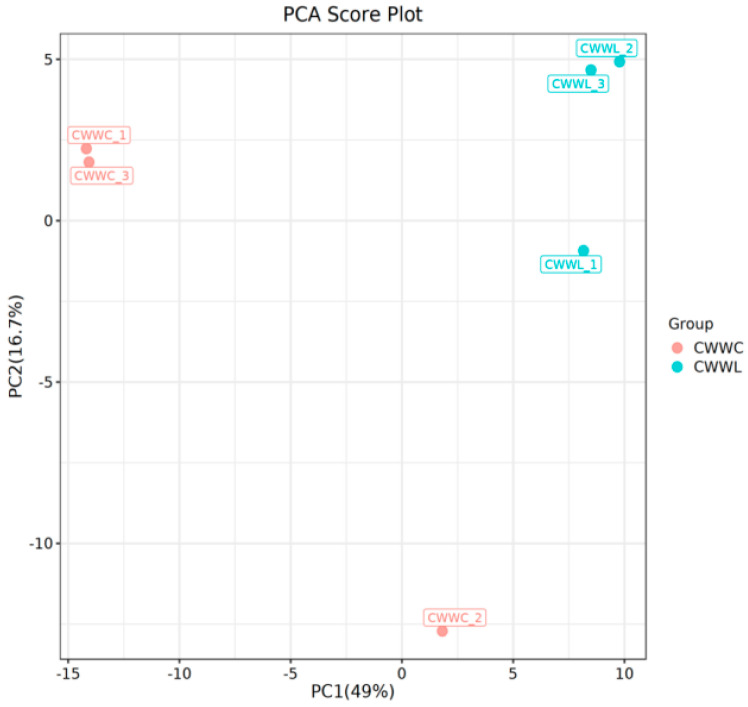
Representative data of PC1 loadings versus PC2 loadings to show the relevance of the culture mode to volatile flavor compounds. Horizontal coordinates indicate the first principal component’s explanatory degree and the second principal component’s explanatory degree in the direction of the horizontal coordinates. Dots indicate samples and colors indicate different groupings.

PCA and OPLS-DA were used to analyze the flavor substances in Chinese longsnout catfish muscles [28]. The PCA revealed significant differences between the samples from the CWWC and CWWL groups (Figure 4). The first principal component (PC1) and the second principal component (PC2) accounted for 49% and 16.7% of the variance, respectively. Meanwhile, the samples in the two groups were significantly different since the CWWC samples were spatially grouped on the left side of PC1 with large intra-individual variability, while the CWWL samples clustered on the right, underscoring distinct aroma component differences.

**Figure 5:**
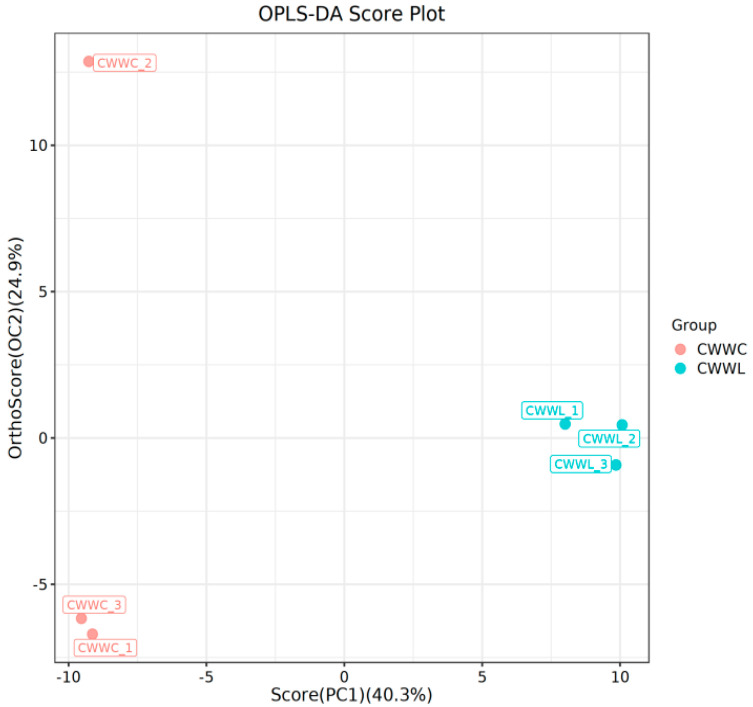
Map of the OPLS-DA scores for both culture modes. Horizontal coordinates indicate the first principal component’s explanatory degree and the second principal component’s explanatory degree in the direction of the horizontal coordinates. Dots indicate samples and colors indicate different groupings.

In addition, an OPLS-DA model was developed to assess the differences in metabolite expression between the two groups. The model exhibited excellent fit and predictive reliability with high R2Y (0.995) and Q2 (0.915) values (Figure 5), indicating a low likelihood of overfitting. VIP values were used to evaluate the significance of the variables within the model. Any variable with VIP > 1 was considered a potential biomarker due to its significant contribution to the model. Using the criteria of VIP > 1 and *p* < 0.05 for the OPLS-DA, we identified 52 significantly differentially expressed metabolites. Among them, tetradecanoic acid displayed the highest VIP value, suggesting its critical role in distinguishing between the two groups. Except that, the other top five variable metabolites included isopropyl palmitate, cyclohexane (2-ethyl-1-methylbutyrolactone), 2-methyl-1-butyramide, 1,7-dimethylnaphthalene, and 3,7,11-trimethyl-1-dodecanol.

#### 3.4.4. Comparison of Differential Metabolites

**Figure 6:**
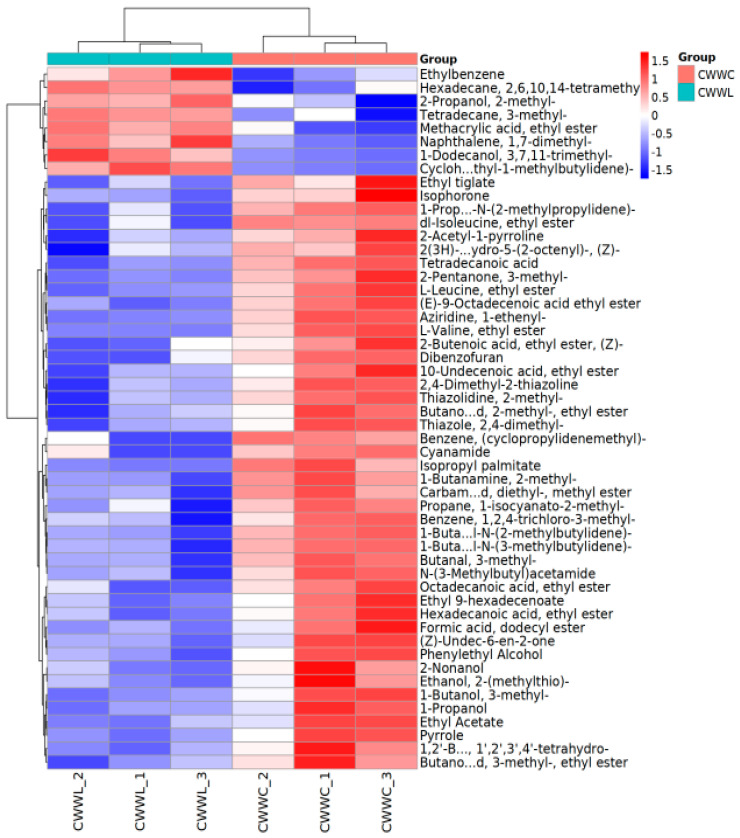
Heat map for clustering of differential compounds. Columns represent samples, rows represent substances, and the clustering tree on the left side of the figure shows the clustering of different substance types.

A differential metabolite analysis revealed notable differences in the abundance of specific volatile compounds between the two groups. In comparison with the CWWC group, fish samples in the CWWL group showed significantly higher levels of eight compounds, including tert-butanol, ethylbenzene, and 3-methyltetradecane (Figure 6), which may contribute to a sweet, fruity, and green flavor, respectively. Conversely, the fish samples in the CWWC group had a higher level of tert-butanol, which gave off a sweet wine aroma. Additionally, the profiles of 44 volatile compounds were significantly higher in the CWWC group than those in the CWWL group (see Figure 6).

**Figure 7:**
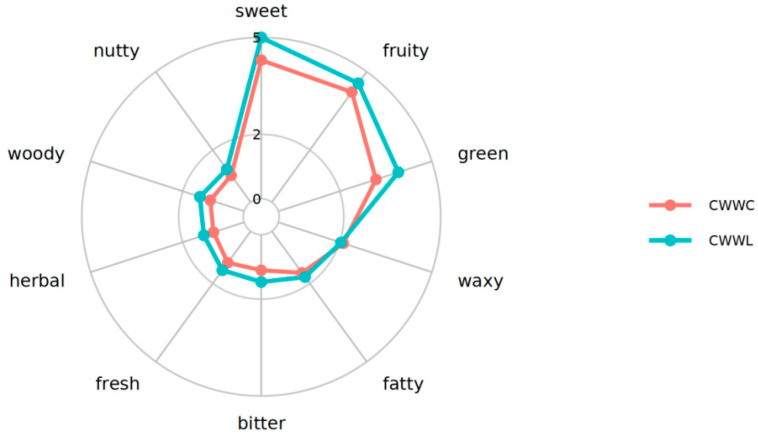
Radar plot of relative content of substance types. The name in the outermost circle indicates the species, the broken line indicates the relative content of the species, and the color indicates the different groupings.

A cluster analysis of the annotated VOCs from the fish samples from two culture modes was conducted by comparing relative odor values with an online flavor database. Our results highlighted that the fish samples in the CWWL group were predominantly preferred to have a sweet, fruity, and green flavor, with secondary preference for fat, woody, herbaceous, fresh, and nutty flavors (Figure 7). Consistently, similar characteristics were also observed in the CWWC group, but it showed relatively lower values of flavor preference (Figure 7). Distinctly, 17 compounds were unique to the CWWL group, such as benzyl alcohol and β-stigmasterol, which contributed to the floral, fruity, and woody flavor, and they enhanced the overall freshness degree. In contrast, the fish samples in the CWWC group contained different compounds, like 2-methylpentane and 2-tridecadecanone, which were associated with an irksome gasoline, earthy, and musty flavor.

**Figure 8:**
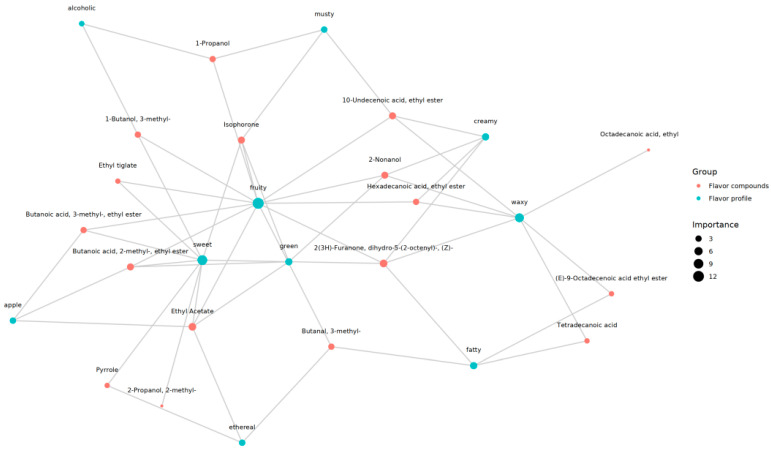
Correlation network diagram between sensory flavor characteristics and flavor substances. Green circles indicate sensory characteristics and red circles indicate flavor compounds.

A correlation network diagram was created to elucidate the relationships between key aroma components, odor classes, and flavors in the fish cultured under both modes. Notably, 2-pentylfuran and 2-ethylhexanol showed significantly higher levels in the CWWL group and may play crucial roles in defining the distinctive aroma of the fish (Figure 8). These components are likely the primary drivers of the observed difference in odor between the two culture modes. Despite the variance in major components, three compounds, namely 2-methylbutanal, 2-undecane, and 2,3-butanedione, had the most significant influence on aroma in the fish cultured under both modes (see Figure 8).

## 4. Discussion

### 4.1. Effects of Culture Mode on Nutritional Composition of Chinese Longsnout Catfish

Fish meat is the main edible part, and it represents the primary nutritional component of fish, whose nutritional composition significantly affects overall muscle quality [29]. The main aim of this study was to investigate the systematic effects of each integrated system, CWWC (pond culture) or CWWL (recirculating water culture), on the muscle nutrients and volatile compounds of the cultured Chinese longsnout catfish. Chinese longsnout catfish cultured in the CWWL group exhibited a lower lipid content and higher protein level compared to the CWWC group. This phenomenon is synergistically driven by multiple environmental factors. For example, constant temperature promotes protein accumulation by maintaining metabolic homeostasis and reducing stress energy consumption [30]; a high water exchange rate accelerates the excretion of metabolic waste such as ammonia and nitrogen, reduces oxidative stress, and inhibits the activation of adipose synthetase to reduce fat deposition; and high dissolved oxygen levels optimize energy metabolism by activating the PPARα pathway to prioritize the mobilization of fat for energy supply and inhibit lipid synthesis in the muscles [31]. Similar results were also obtained in grass carp [32] and largemouth bass [33] cultured in flow-through tank systems, where enhanced exercise could stimulate lipid metabolism and concurrently elevate muscle protein content. Indeed, fish cultured in an RAS environment often show a high ability to move, which improves metabolic rates and energy expenditure in Chinese longsnout catfish, resulting in glycogen depletion and lipid utilization for energy demand.

### 4.2. Effects of Culture Mode on Amino Acid Composition of Chinese Longsnout Catfish

The nutritional value of fish is dependent on their amino acid content and the proportion of EAAs [34]. Our findings demonstrated that both the AA and EAA levels of the fish in the CWWL group were significantly higher than those of the fish in the CWWC group, suggesting that the Chinese longsnout catfish cultured in the recirculating water had higher nutritional value in comparison with those cultured in traditional ponds. Moderate current stimulation of fish movement can enhance muscle vitality through ‘exercise-induced protein synthesis’ to promote the development of muscle fibers and increase muscle protein content; at the same time, the current accelerates the diffusion of nutrients, such as feed proteins, in the water column, thereby improving feeding efficiency and digestion and absorption rates. Our results are consistent with a study on mackerel (*Spinibarbus sinensis*) cultured in a running water system [35], implying that recirculating water culture is indeed a better culture mode for improving the nutritional value of targeted fish. Meanwhile, according to the standard for high-quality protein established by the FAO/WHO, the EAA/TAA ratio should be approximately 40% and the EAA/NEAA ratio should be about 60% [36]. In our current study, the EAA/TAA ratios of Chinese longsnout catfish cultured in both culture modes were 40.5% and 40.7%, and the EAA/NEAA ratios were 80.5% and 81.1%, respectively, confirming that Chinese longsnout catfish are a high-quality protein source, and their nutritional value is even superior to that of rainbow trout [37] and tuna [38] products.

### 4.3. Effects of Culture Mode on Fatty Acid Composition of Chinese Longsnout Catfish

The fatty acid composition and content significantly influence the flavor and quality of food [39]. SFAs, a primary energy resource for organisms, are the dominant fatty acids identified in Chinese longsnout catfish. Among them, palmitic acid (C16:0) was the main component in fish cultured under both modes. Interestingly, the ΣSFA level of the fish sample in the CWWL group was significantly higher than that of the fish in the CWWC group, implying that fish cultured in a recirculating water environment require more energy supplementation and thus an elevated SFA content can meet their corresponding energy demand. PUFAs, which are key indicators of fatty acid nutritional value, are well known for their health benefits, including maintaining cell membrane fluidity, regulating fat metabolism, enhancing immunity, and reducing inflammation [40]. Among them, EPAs and DHAs are particularly crucial for infant brain development, cardiovascular health, and cancer growth modulation [41]. The content of Σn-6 and Σn-3 fatty acids was significantly higher in the CWWL group, with the EPA and DHA levels being nearly two-fold greater that in the CWWC group, supporting the higher nutritional value of the fish cultured in recirculating water [42]. Conversely, the ΣMUFA content of the fish in the CWWC group was significantly higher. These differences are strongly linked to environmental factors other than diet, such as the water temperature, water depth, water flow velocity, and variable microorganism components in the culture system [43]. The microbial diversity of traditional ponds is usually low, mostly dominated by anaerobic bacteria, whose metabolites (such as ammonia and hydrogen sulfide) inhibit lipid digestive enzyme activity, resulting in a decrease in fatty acid uptake in the CWWC group. In summary, Chinese longsnout catfish cultured in the recirculating water system exhibited superior compatibility with human dietary demands and had enhanced nutritional quality in comparison with those cultured in ponds. Similar findings were also reported for largemouth bass (*Micropterus nigricans*) [44], underscoring the influence of aquaculture conditions on fatty acid profiles and the resultant meat quality.

### 4.4. Effects of Culture Mode on Volatile Flavor Compounds of Chinese Longsnout Catfish

Peroxidase and lipoxygenase are two well-known enzymes that influence the production of volatile compounds in aquaculture fish under various environmental conditions [45]. Generally speaking, aldehydes and ketones are primary volatile compounds that contribute to flavor, originating from microbial denaturation and the autoxidation of amino acids and fatty acids [46]. Aldehydes can significantly enhance the flavor of Chinese longsnout catfish, possibly due to their lower sensory thresholds and the presence of some common volatile aldehydes, such as nonanal, heptanal, decanal, and octanal [47]. Notably, octanal and nonanal are oxidation products of oleic acid, and they usually lead to grassy and fatty flavors in freshwater fish. Meanwhile, 2-methylbutyraldehyde originates from the Strecker degradation of isoleucine, which confers a pronounced nutty flavor [48]. It is usually detected in various aquatic products, including large yellow croaker (*Larimichthys crocea*) [49] and Peruvian squid (*Dosidicus gigas*) [50], as well as in foods such as ham [51] and sausage [52]. Ketones are typical products of the thermal oxidation of polyunsaturated fatty acids, amino acid catabolism, or microbial oxidation and are well known for their creamy and fruity flavors [53]. Furthermore, 2,3-butanedione was identified as the predominant volatile flavor compound under both culture modes, and it contributed a creamy and fruity flavor [54]. In contrast, isophorone is associated with undesirable minty, camphorated, and musty flavors. Additionally, dimethyl trisulfide, noted for its sulfuric, onion-like, spicy, and salty flavors, was only detected in the pond-cultured fish. Pond sediments are rich in organic matter and are anaerobic for a long period of time, and they are prone to breeding anaerobic and spoilage bacteria [55], which affect flavor through metabolic pathways: in lipid metabolism, the lipolytic enzymes secreted by them break down muscle fat, and free fatty acids are oxidized by lipoxygenase to produce octanal, nonanal, and isophorone, while in amino acid metabolism, the Strecker degradation of isoleucine leads to the decomposition of sulfur-containing amino acids to produce the distinctive dimethyl trisulphide of the CWWC group. In conclusion, these findings suggest that fish muscles from the recirculating water culture group exhibit a more favorable flavor profile compared to those from the pond culture group.

In this study, we observed significantly higher ROAVs in the CWWL group, indicating that the culture mode has a significant effect on volatile flavor compound compositions as well as flavor compound profiles. Meanwhile, the fish cultured in the CWWL group contained higher levels of 2-methylbutyraldehyde, 2,3-butanedione, 2-pentylfuran, and 2-undecane, which may produce prominent odors reminiscent of cocoa powder, almonds, and butter, along with notes of citrus, freshness, green beans, and vegetables. Our fish from the CWWC group contained small amounts of 2-methylbutyraldehyde, 2-undecane, 2,3-butanedione, isophorone, and dimethyl trisulfide, which may have contributed to a range of odors, from sharp and malodorous to oniony and pungent scents. These findings show that the volatile flavor of fish cultured in recirculating water is much better, and this culture mode should be promoted worldwide.

## 5. Conclusions

This study demonstrated that Chinese longsnout catfish cultured in a circulating water system exhibited better nutritional and sensory properties than those cultured in a pond. In general, the CWWL group displayed higher levels of proteins and lower levels of lipids. Notably, amino acids, including NEAAs, EAAs, and DAAs, were also significantly more abundant in the CWWL group, resulting in enhanced fresh and sweet flavors. Meanwhile, the levels of EPAs and DHAs were significantly higher in the CWWL group, implying that Chinese longsnout catfish have higher nutritional value when cultured in a recirculating water environment. Additionally, the diversity and palatability of favorable volatile flavor compounds, including 2-methylbutyraldehyde and 2,3-butanedione, were markedly richer in the fish cultured in a circulating water environment. Conversely, the fish cultured in a pond contained high levels of volatile compounds, such as 2-tridecanone, dimethyl trisulfide, and isophorone, which may contribute to an unfavorable sensory flavor. The shortcoming of this study is the small sample size, but we maximized the reliability and credibility of the study through rigorous experimental design, multi-dimensional analyses and validation, and reasonable statistical methods. In conclusion, our findings prove that the nutritional value and favorable volatile flavor of Chinese longsnout catfish cultured in a circulating water system are significantly better than those of Chinese longsnout catfish cultured in a pond, which supports the potential benefits of circulating water aquaculture systems in producing high-quality and flavorful fish products.

## Figures and Tables

**Table 1:** Basic nutritional contents of Chinese longsnout catfish under two culture modes.

Items	CWWC	CWWL
Ash (%)	1.0 ± 0.09	1.10 ± 0.1
Moisture (%)	78.6 ± 0.08 ^a^	79.4 ± 0.12 ^b^
Crude protein (%)	16.99 ± 0.13 ^a^	17.7 ± 0.05 ^b^
Crude lipid (%)	4.40 ± 0.05 ^a^	1.60 ± 0.03 ^b^

CWWC stands for pond culture and CWWL stands for recirculating culture systems; different letters indicate significant differences from the control (CWWC): a, control; b, *p* < 0.05.

**Table 2:** Amino acid composition of Chinese longsnout catfish under two culture modes.

Amino Acid	Content g/100 g	
	CWWC	CWWL
Thr	0.76 ± 0.01 ^a^	0.84 ± 0.02 ^b^
Val	0.73 ± 0.01	0.79 ± 0.04
Met	0.47 ± 0.04	0.51 ± 0.02
Phe	0.67 ± 0.03	0.73 ± 0.04
Ile	0.72 ± 0.03	0.77 ± 0.01
Leu	1.32 ± 0.01 ^a^	1.44 ± 0.01 ^b^
Lys	1.58 ± 0.03 ^a^	1.70 ± 0.01 ^b^
Asp	1.70 ± 0.05 ^a^	1.86 ± 0.03 ^b^
Glu	2.49 ± 0.04 ^a^	2.72 ± 0.02 ^b^
Gly	0.74 ± 0.01	0.75 ± 0.04
Ala	0.91 ± 0.02	0.97 ± 0.04
Ser	0.68 ± 0.03 ^a^	0.74 ± 0.01b
Tyr	0.61 ± 0.04	0.66 ± 0.01
Cys	0.13 ± 0.02	0.13 ± 0.03
Pro	0.50 ± 0.05	0.53 ± 0.01
His	0.42 ± 0.04	0.45 ± 0.04
Arg	1.0 ± 0.04	1.07 ± 0.03
Total ∑TAA	15.43 ± 0.23 ^a^	16.66 ± 0.10 ^b^
Total ∑EAA	6.25 ± 0.05 ^a^	6.78 ± 0.10 ^b^
Total ∑NEAA	7.76 ± 0.19 ^a^	8.36 ± 0.08 ^b^
Total ∑HEAA	1.42 ± 0.04	1.52 ± 0.07
Total ∑DAA	5.84 ± 0.10 ^a^	6.3 ± 0.08 ^b^

CWWC stands for pond culture and CWWL stands for recirculating culture systems, with letters labeling differences (a: CWWC control; b: significant difference; unlabeled: no difference). Thr: threonine; Val: valine; Met: methionine; Phe: phenylalanine; Ile: isoleucine; Leu: leucine; Lys: lysine; Asp: aspartic acid; Glu: glutamic acid; Ala: alanine; Ser: serine; Tyr: tyrosine; Cys: cysteine; Pro: proline; His: histidine; Arg: arginine. TAAs: total amino acids; EAAs: essential amino acids; NEAAs: non-essential amino acids; HEAAs: half essential amino acids; DAAs: delicious amino acids.

**Table 3:** Fatty acid composition of Chinese longsnout catfish under two culture modes.

Fatty Acid	Content 100%	
	CWWC	CWWL
C12:0	0.04 ± 0.01	0.03 ± 0.01
C14:0	1.46 ± 0.06 ^a^	2.46 ± 0.05 ^b^
C15:0	0.18 ± 0.04 ^a^	0.29 ± 0.04 ^b^
C16:0	19.3 ± 0.14 ^a^	20.30 ± 0.07 ^b^
C17:0	0.32 ± 0.04 ^a^	0.56 ± 0.06 ^b^
C18:0	5.54 ± 0.05	5.58 ± 0.07
C20:0	0.27 ± 0.02	0.23 ± 0.04
C14:1n5	0.03 ± 0.02 ^a^	0.07 ± 0.02 ^b^
C16:1n7	3.49 ± 0.07 ^a^	5.01 ± 0.10 ^b^
C17:1n7	0.31 ± 0.02 ^a^	0.47 ± 0.03
C18:1n9c	39.00 ± 0.14 ^a^	31.10 ± 0.17 ^b^
C22:1n9	0.17 ± 0.03	0.14 ± 0.03
C18:2n6c	16.70 ± 0.07 ^a^	14.10 ± 0.03 ^b^
C18:3n6	0.09 ± 0.03	0.12 ± 0.03
C18:3n3	1.39 ± 0.04 ^a^	1.70 ± 0.03 ^b^
C20:2	1.06 ± 0.07	0.98 ± 0.10
C20:3n6	0.50 ± 0.05 ^a^	0.75 ± 0.12 ^b^
C20:3n3	0.19 ± 0.02	0.24 ± 0.09
C20:4n6 (AA)	0.71 ± 0.02 ^a^	1.44 ± 0.06 ^b^
C22:2n6	0.03 ± 0.01 ^a^	0.16 ± 0.03 ^b^
C20:5n3 (EPA)	1.40 ± 0.07 ^a^	2.50 ± 0.03 ^b^
C22:6n3 (DHA)	4.74 ± 0.05 ^a^	9.09 ± 0.05 ^b^
ΣSFA	27.11 ± 0.08 ^a^	32.25 ± 0.06 ^b^
ΣMUFA	43.00 ± 0.13 ^a^	36.79 ± 0.12 ^b^
ΣPUFA	26.81 ± 0.04 ^a^	31.07 ± 0.15 ^b^
EPA + DHA	6.14 ± 0.06 ^a^	11.59 ± 0.02 ^b^

CWWC stands for pond culture and CWWL stands for recirculating culture systems, with letters labeling differences (a: CWWC control; b: significant difference; unlabeled: no difference). C12:0: lauric acid; C14:0: myristic acid; C15:0: pentadecanoic acid; C16:0: palmitic acid; C17:0: heptadecanoic acid; C18:0: stearic acid; C20:0: arachidic acid; C14:1n5: cis-9-tetradecenoic acid; C16:1n7: cis-9-hexadecenoic acid; C17:1n7: cis-10-heptadecenoic acid; C18:1n9c: oleic acid; C22:1n9: erucic acid; C18:2n6c: linoleic acid; C18:3n6: y-linolenic acid; C18:3n3: a-linolenic acid; C20:2: cis-11,14-eicosadienoic acid; C20:3n6: cis-8,11,14-eicosatrienoic acid; C20:3n3: cis-11,14,17-eicosatrienoic acid; C20:4n6 (AA): arachidonic acid; C22:2n6: cis-13,16-docosadienoic acid; EPA: eicosapentaenoic acid; DHA: docosahexaenoic acid; SFAs: saturated fatty acids; MUFAs: monounsaturated fatty acids; PUFAs: polyunsaturated fatty acids.

## Data Availability

The original contributions presented in this study are included in the article. Further inquiries can be directed to the corresponding author(s).

## Supplementary Materials

The following supporting information can be downloaded at https://www.mdpi.com/article/10.3390/biology14060694/s1, Table S1. A list of various metabolites in fishes from both culture modes.
