# Supplementary material for: Effects of Two Culture Modes on Muscular Nutrition Content and Volatile Flavor in Chinese Longsnout Catfish (Leiocassis longirostris)
Source: Biology (Basel). 2025 Jun 13;14(6):694. doi: 10.3390/biology14060694 (PMC12189077; doi:10.3390/biology14060694)
Supplement: Supplementary file 1 [file biology-14-00694-s001.zip › biology-3641787-supplementary.pdf]

Table S1 A list of various metabolites in fishes from both culture modes

Name	CAS	Formula	log2FC	P.value	-log10(P.value)	VIP
Tetradecanoic acid	544-63-8	C14H28O2	-2.08	0.001775379	2.75	1.533158509
Isopropyl palmitate	142-91-6	C19H38O2	-3.43	0.012224308	1.91	1.522237442
2-methyl-1-Butanamine	96-15-1	C5H13N	-7.12	0.00199086	2.7	1.51840564
1,7-dimethyl-Naphthalene,	575-37-1	C12H12	2.07	0.003938218	2.4	1.516748428
3,7,11-trimethyl-1-Dodecanol	6750-34-1	C15H32O	3.29	0.002347005	2.63	1.511713034
2-methyl-N-(2-methylbutylidene)-1-Butanamine	54518-97-7	C10H21N	-7.02	0.003964964	2.4	1.500934456
1-ethenyl-Aziridine	5628-99-9	C4H7N	-6.55	0.003372484	2.47	1.499171014
3-methyl-2-Pentanone	565-61-7	C6H12O	-2.6	0.004676164	2.33	1.496355447
2-methyl-N-(2-methylpropylidene)-1-Propanamine	6898-82-4	C8H17N	-7.14	0.009369191	2.03	1.485969412
L-Valine, ethyl ester	17431-03-7	C7H15NO2	-4.08	0.02944242	1.53	1.484987658
L-Leucine, ethyl ester	2743-60-4	C8H17NO2	-3.73	0.005688054	2.25	1.482366289
Carbamodithioic acid, diethyl-, methyl ester	686-07-7	C6H13NS2	-2.47	0.006391625	2.19	1.481164693
dl-Isoleucine, ethyl ester	55056-62-7	C8H17NO2	-4.47	0.045126286	1.35	1.480887167
3-methyl-Butanal	590-86-3	C5H10O	-5.5	0.006798977	2.17	1.479251989
3-methyl-N-(3-methylbutylidene)-1-Butanamine	35448-31-8	C10H21N	-8.03	0.006863354	2.16	1.478003007
(E)-9-Octadecenoic acid ethyl ester	6114-18-7	C20H38O2	-4.05	0.004809456	2.32	1.470726006
N-(3-Methylbutyl) acetamide	13434-12-3	C7H15NO	-6.7	0.013770197	1.86	1.438226655
2-methyl-Thiazolidine	24050-16-6	C4H9NS	-5.1	0.015808468	1.8	1.434934319
2,4-Dimethyl-2-thiazoline	6114-40-5	C5H9NS	-5.85	0.019642368	1.71	1.413816227
2,4-dimethyl-Thiazole	541-58-2	C5H7NS	-5.15	0.020891755	1.68	1.397840178
1-isocyanato-2-methyl-Propane	1873-29-6	C5H9NO	-4.75	0.02670627	1.57	1.39614245
Ethyl tiglate	5837-78-5	C7H12O2	-1.69	0.027524188	1.56	1.391886238
Butanoic acid, 3-methyl-, ethyl ester	108-64-5	C7H14O2	-1.53	0.020542839	1.69	1.386628971
Methacrylic acid, ethyl ester	97-63-2	C6H10O2	1.97	0.023712003	1.63	1.385424932
2-Acetyl-1-pyrroline	85213-22-5	C6H9NO	-4.85	0.028678783	1.54	1.385221218
3-methyl-1-Butanol	123-51-3	C5H12O	-3.14	0.021423025	1.67	1.38511789
Pyrrole	109-97-7	C4H5N	-3.28	0.020453268	1.69	1.373944092
Phenylethyl Alcohol	60-12-8	C8H10O	-1.65	0.024606277	1.61	1.36969787
Dibenzofuran	132-64-9	C12H8O	-3.69	0.020376611	1.69	1.368965859
(cyclopropylidenemethyl)-Benzene	7555-67-1	C10H10	-5.2	0.01807361	1.74	1.365112569
3-methyl-Tetradecane	18435-22-8	C15H32	2.77	0.028126028	1.55	1.365024044
Hexadecanoic acid, ethyl ester	628-97-7	C18H36O2	-3.4	0.021929673	1.66	1.363099709
1',2',3',4'-tetrahydro-1,2'-Binaphthalene	32675-22-2	C20H18	-2.64	0.021702765	1.66	1.362119139
2,6,10,14-tetramethyl-Hexadecane	638-36-8	C20H42	4.54	0.024941301	1.6	1.360823585
Octadecanoic acid, ethyl ester	111-61-5	C20H40O2	-5.36	0.022376616	1.65	1.356893009
1,2,4-trichloro-3-methyl-Benzene	2077-46-5	C7H5Cl3	-3.15	0.031670949	1.5	1.354362482
Isophorone	78-59-1	C9H14O	-2.44	0.032023625	1.49	1.350645311
dihydro-5-(2-octenyl)-, (Z)-2(3H)-Furanone	18679-18-0	C12H20O2	-6.02	0.040886858	1.39	1.349757569
Butanoic acid, 2-methyl-, ethyl ester	7452-79-1	C7H14O2	-1.95	0.033087282	1.48	1.34870806
10-Undecenoic acid, ethyl ester	692-86-4	C13H24O2	-6.67	0.037027865	1.43	1.341471391
Ethylbenzene	100-41-4	C8H10	3.3	0.03030504	1.52	1.338747074
Ethyl 9-hexadecenoate	54546-22-4	C18H34O2	-2.67	0.028682921	1.54	1.334596368
2-Nonanol	628-99-9	C9H20O	-3.21	0.030927967	1.51	1.325402892
Formic acid, dodecyl ester	28303-42-6	C13H26O2	-3.75	0.040533788	1.39	1.324644845
1-Propanol	71-23-8	C3H8O	-2.99	0.042599444	1.37	1.307013578
2-methyl-2-Propanol	75-65-0	C4H10O	3.13	0.049992111	1.3	1.296009384
(Z)-Undec-6-en-2-one	107853-70-3	C11H20O	-4.06	0.047041976	1.33	1.293820319
Cyanamide	420-04-2	CH2N2	-4.67	0.036967497	1.43	1.28658915
(Z)-2-Butenoic acid, ethyl ester	6776-19-8	C6H10O2	-2.01	0.043545493	1.36	1.285409054
Ethyl Acetate	141-78-6	C4H8O2	-3.59	0.044975396	1.35	1.281491636
2-(methylthio)-Ethanol	5271-38-5	C3H8OS	-2.18	0.047719534	1.32	1.273020071
